# Supplementary figures and images for: Prognostic value of postoperative decrease in serum albumin on surgically resected early-stage non-small cell lung carcinoma: A multicenter retrospective study
Source: PLoS One. 2021 Sep 2;16(9):e0256894. doi: 10.1371/journal.pone.0256894 (PMC8412276; doi:10.1371/journal.pone.0256894)

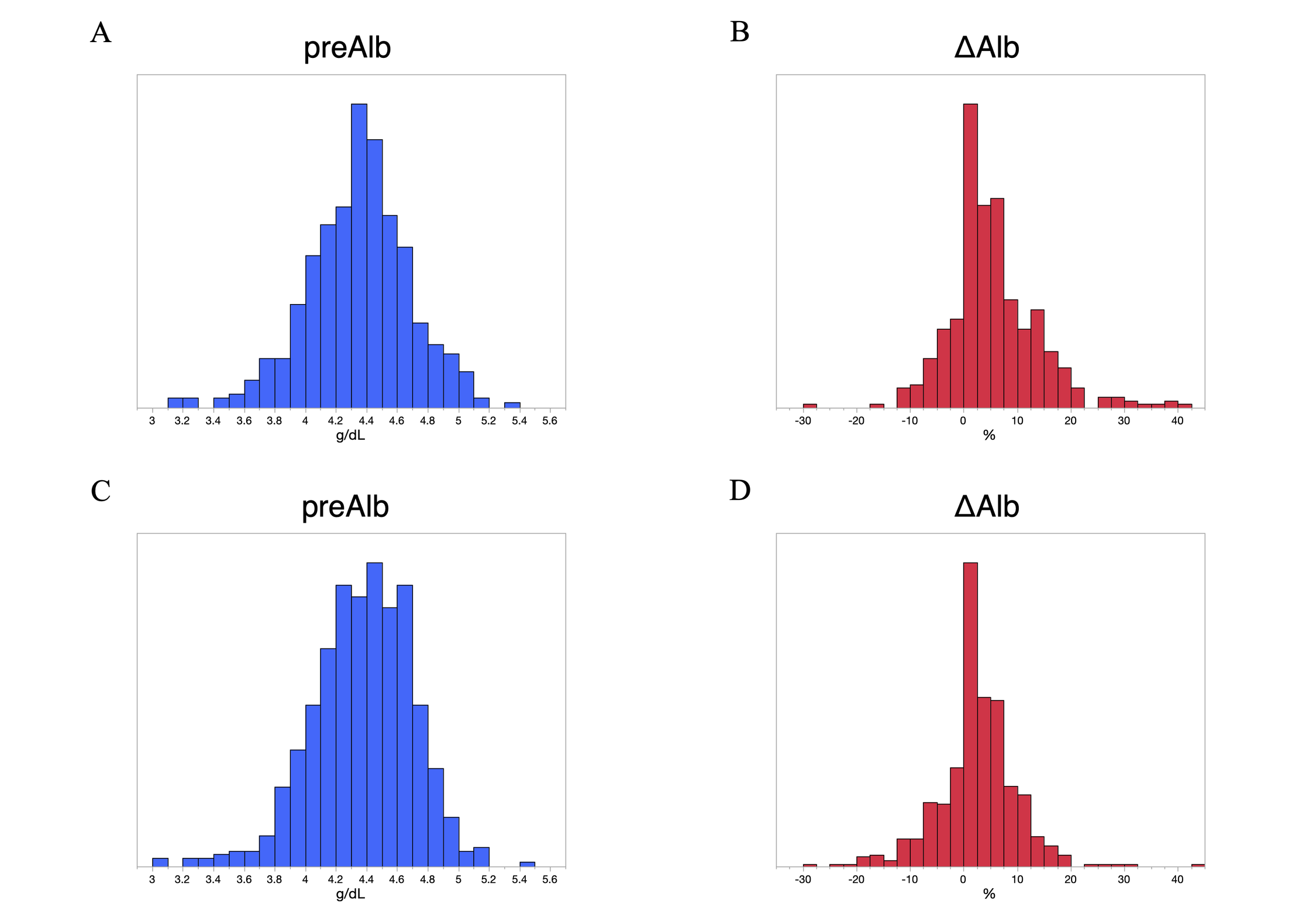

Supplement: S1 Fig — The histograms of distributions of preAlb and ΔAlb in the training cohort (A, B) and in the validation cohort (C, D). (TIF) [file pone.0256894.s001.tif]

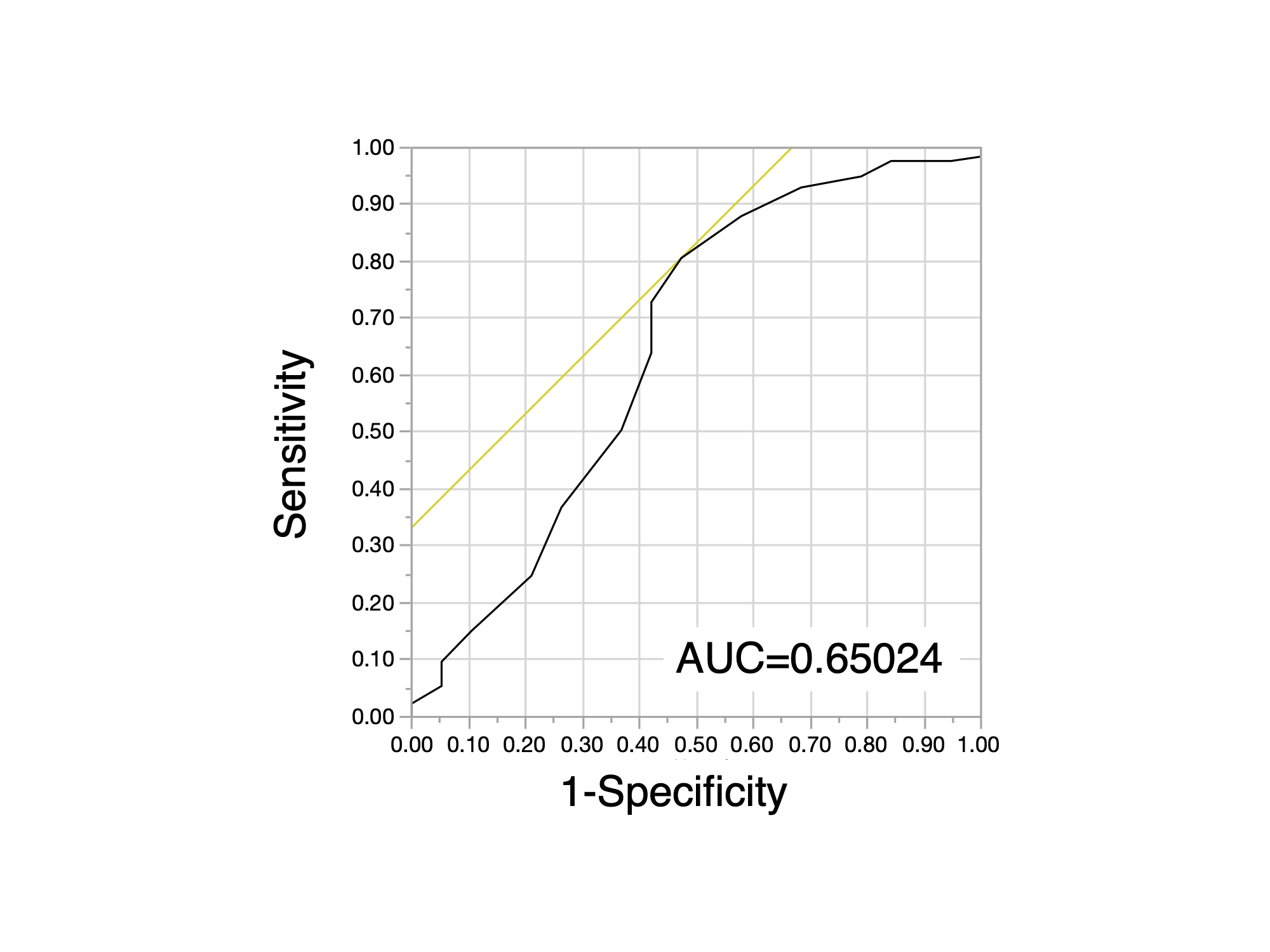

Supplement: S2 Fig — ROC curves for preoperative serum albumin levels (preAlb) in the TC. AUC: area under the curve. (TIF) [file pone.0256894.s002.tif]

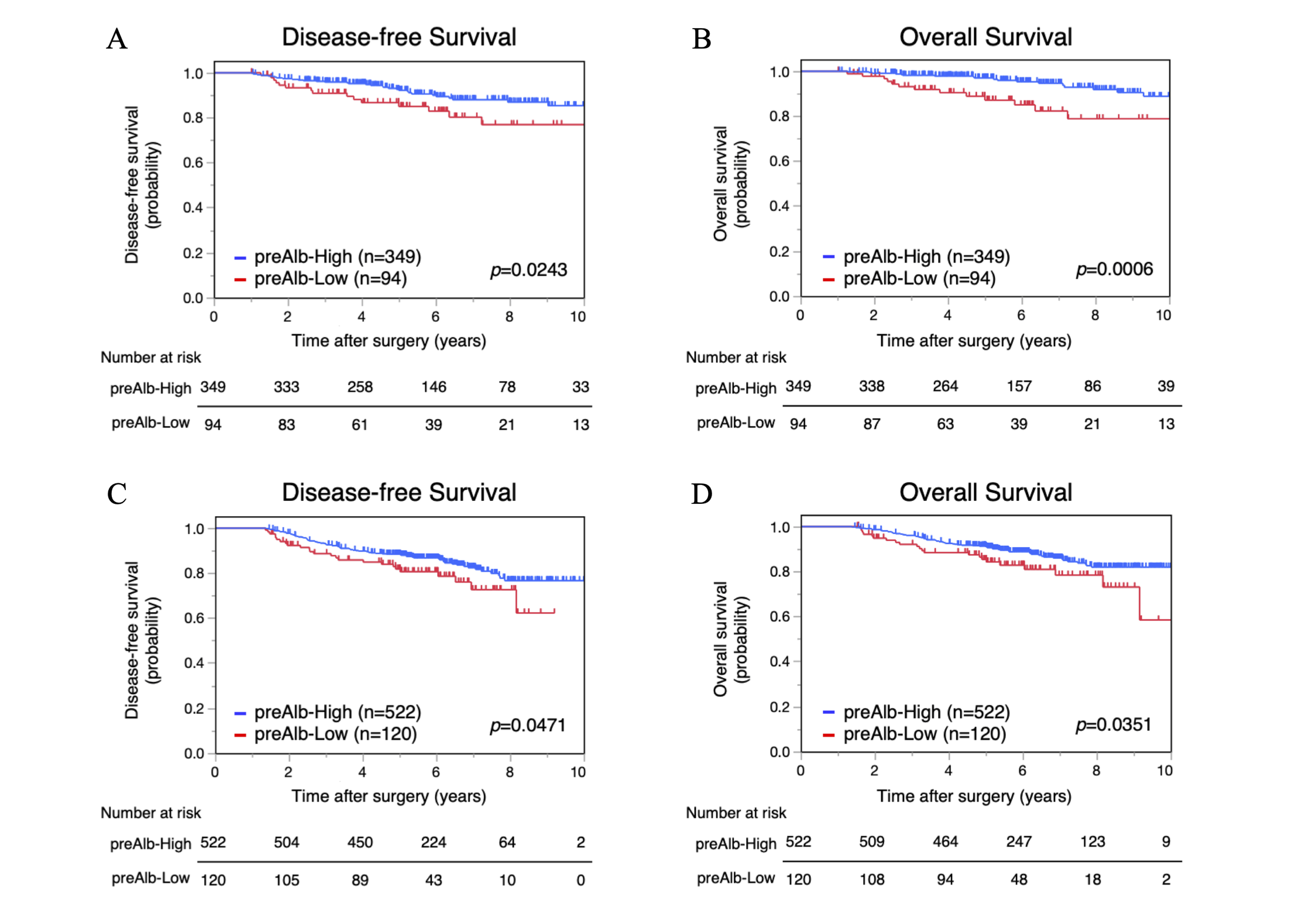

Supplement: S3 Fig — Disease-free survival and overall survival of the preAlb-Low and preAlb-High groups in the training cohort (A, B) and in the validation cohort (C, D). (TIF) [file pone.0256894.s003.tif]

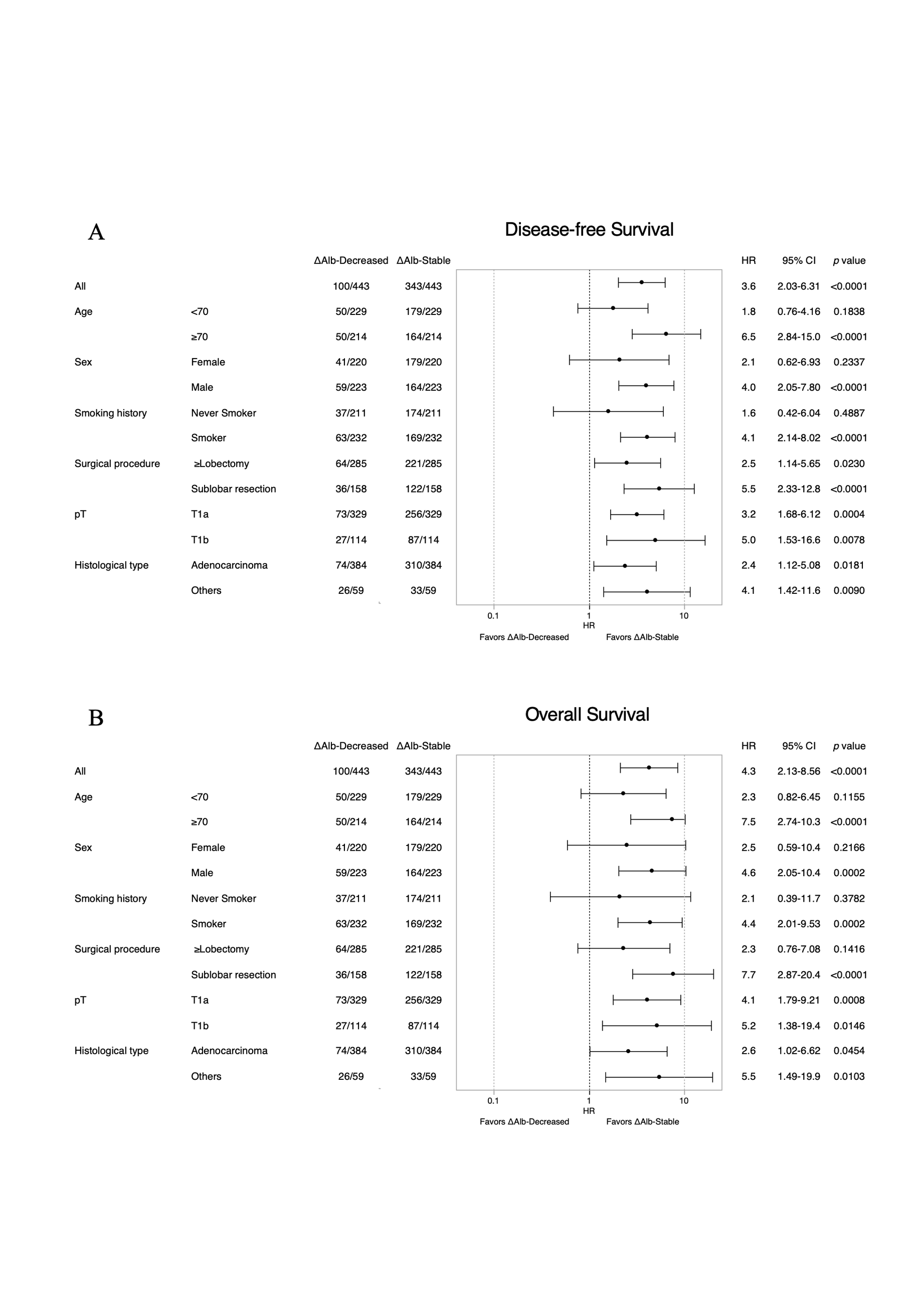

Supplement: S4 Fig — Hazard ratios of ΔAlb for (A) disease-free survival and (B) overall survival in the TC. HR: hazard ratio, CI: confidence interval. (TIF) [file pone.0256894.s004.tif]
