# Supplementary material for: Prognostic value of postoperative decrease in serum albumin on surgically resected early-stage non-small cell lung carcinoma: A multicenter retrospective study
Source: PLoS One. 2021 Sep 2;16(9):e0256894. doi: 10.1371/journal.pone.0256894 (PMC8412276; doi:10.1371/journal.pone.0256894)
Supplement: S1 Table — (DOCX) [file pone.0256894.s005.docx]

**Supplementary Table 1**. Clinicopathological characteristics of patients with pStage IA non-small cell lung carcinoma

| Characteristics |  | Training cohort (n=443) | | | Validation cohort (n=642) | |
| --- | --- | --- | --- | --- | --- | --- |
| Age, years | <70 | 229 | (51.7%) |  | 363 | (56.5%) |
|  | ≥70 | 214 | (48.3%) |  | 279 | (43.5%) |
| Sex | Female | 220 | (49.7%) |  | 359 | (55.9%) |
|  | Male | 223 | (50.3%) |  | 283 | (44.1%) |
| Smoking | Never smoker | 211 | (47.6%) |  | 347 | (54.0%) |
|  | Smoker | 232 | (52.4%) |  | 295 | (46.0%) |
| Pulmonary comorbidity | Absent | 425 | (95.9%) |  | 605 | (94.2%) |
|  | Present | 18 | (4.1%) |  | 37 | (5.8%) |
| Surgical procedure | ≥Lobectomy | 285 | (64.3%) |  | 457 | (71.2%) |
|  | Sublobar resection | 158 | (35.7%) |  | 185 | (28.8%) |
| pT | T1a | 329 | (74.3%) |  | 443 | (69.0%) |
|  | T1b | 114 | (25.7%) |  | 199 | (31.0%) |
| Histological type | Adenocarcinoma | 384 | (86.7%) |  | 571 | (88.9%) |
|  | Non-adenocarcinoma | 59 | (13.3%) |  | 71 | (11.1%) |
| Vascular invasion | Negative | 402 | (90.7%) |  | 625 | (97.4%) |
|  | Positive | 41 | (9.3%) |  | 17 | (2.6%) |
| Lymphatic invasion | Negative | 433 | (97.7%) |  | 599 | (93.3%) |
|  | Positive | 10 | (2.3%) |  | 43 | (6.7%) |
| ΔAlb | Stable | 343 | (77.4%) |  | 584 | (91.0%) |
|  | Decreased | 100 | (22.6%) |  | 58 | (9.0%) |
| preAlb | High | 349 | (78.8%) |  | 522 | (81.3%) |
|  | Low | 94 | (21.2%) |  | 120 | (18.7%) |

pStage, pathological stage; pT, pathological T status; ΔAlb, postoperative decrease in serum albumin; preAlb, preoperative serum albumin levels
