# Supplementary material for: Prognostic value of postoperative decrease in serum albumin on surgically resected early-stage non-small cell lung carcinoma: A multicenter retrospective study
Source: PLoS One. 2021 Sep 2;16(9):e0256894. doi: 10.1371/journal.pone.0256894 (PMC8412276; doi:10.1371/journal.pone.0256894)
Supplement: S2 Table — (DOCX) [file pone.0256894.s006.docx]

**Supplementary Table 2**. Associations of clinicopathological characteristics with preAlb

|  |  | Training cohort | | | | |  | Validation cohort | | | | |
| --- | --- | --- | --- | --- | --- | --- | --- | --- | --- | --- | --- | --- |
|  |  | preAlb | | | |  |  | preAlb | | | |  |
| Characteristics | | Low (n=94) | | High (n=349) | | *p* value |  | Low (n=120) | | High (n=522) | | *p* value |
| Age, years |  |  |  |  |  |  |  |  |  |  |  |  |
| <70 |  | 31 | (33.0%) | 198 | (56.7%) | <0.0001 |  | 40 | (33.3%) | 323 | (61.9%) | <0.0001 |
| ≥70 |  | 63 | (67.0%) | 151 | (43.3%) |  |  | 80 | (66.7%) | 199 | (38.1%) |  |
| Sex |  |  |  |  |  |  |  |  |  |  |  |  |
| Female |  | 37 | (39.4%) | 183 | (52.4%) | 0.0244 |  | 64 | (53.3%) | 295 | (56.5%) | 0.5269 |
| Male |  | 57 | (60.6%) | 166 | (47.6%) |  |  | 56 | (46.7%) | 227 | (43.5%) |  |
| Smoking |  |  |  |  |  |  |  |  |  |  |  |  |
| Never smoker |  | 33 | (35.1%) | 178 | (51.0%) | 0.0062 |  | 63 | (52.5%) | 284 | (54.4%) | 0.7056 |
| Smoker |  | 61 | (64.9%) | 171 | (49.0%) |  |  | 57 | (47.5%) | 238 | (45.6%) |  |
| Pulmonary comorbidity | |  |  |  |  |  |  |  |  |  |  |  |
| Absent | | 88 | (93.4%) | 337 | (96.7%) | 0.2358 |  | 113 | (94.2%) | 492 | (94.2%) | 1.0000 |
| Present | | 6 | (6.4%) | 12 | (3.4%) |  |  | 7 | (5.8%) | 30 | (5.8%) |  |
| Surgical procedure | |  |  |  |  |  |  |  |  |  |  |  |
| ≥Lobectomy |  | 59 | (62.8%) | 226 | (64.8%) | 0.7206 |  | 82 | (68.3%) | 375 | (71.8%) | 0.4445 |
| Sublobar resection | | 35 | (37.2%) | 123 | (35.2%) |  |  | 38 | (31.7%) | 147 | (28.2%) |  |
| pT |  |  |  |  |  |  |  |  |  |  |  |  |
| T1a |  | 68 | (72.3%) | 261 | (74.8%) | 0.6304 |  | 77 | (64.2%) | 366 | (70.1%) | 0.2039 |
| T1b |  | 26 | (27.7%) | 88 | (25.2%) |  |  | 43 | (35.8%) | 156 | (29.9%) |  |
| Histological type | |  |  |  |  |  |  |  |  |  |  |  |
| Adenocarcinoma | | 74 | (78.7%) | 310 | (88.8%) | 0.0158 |  | 100 | (83.3%) | 471 | (90.2%) | 0.0359 |
| Non-adenocarcinoma |  | 20 | (21.3%) | 39 | (11.2%) |  |  | 20 | (16.7%) | 51 | (9.8%) |  |
| Vascular invasion | |  |  |  |  |  |  |  |  |  |  |  |
| Negative |  | 82 | (87.2%) | 320 | (91.7%) | 0.1857 |  | 114 | (95.0%) | 511 | (97.9%) | 0.0751 |
| Positive |  | 12 | (12.8%) | 29 | (8.3%) |  |  | 6 | (5.0%) | 11 | (2.1%) |  |
| Lymphatic invasion | |  |  |  |  |  |  |  |  |  |  |  |
| Negative |  | 90 | (95.7%) | 343 | (98.3%) | 0.1418 |  | 106 | (88.3%) | 493 | (94.4%) | 0.0157 |
| Positive |  | 4 | (4.3%) | 6 | (1.7%) |  |  | 14 | (11.7%) | 29 | (5.6%) |  |

preAlb, preoperative serum albumin levels; pT, pathological T status
