# Supplementary material for: Prognostic value of postoperative decrease in serum albumin on surgically resected early-stage non-small cell lung carcinoma: A multicenter retrospective study
Source: PLoS One. 2021 Sep 2;16(9):e0256894. doi: 10.1371/journal.pone.0256894 (PMC8412276; doi:10.1371/journal.pone.0256894)
Supplement: S3 Table — (DOCX) [file pone.0256894.s007.docx]

**Supplementary Table 3.** **Univariable and multivariable analyses of disease-free survival and overall survival in the training cohort**

|  |  |  | Disease-free survival | | | | | | |  | Overall survival | | | | | | |
| --- | --- | --- | --- | --- | --- | --- | --- | --- | --- | --- | --- | --- | --- | --- | --- | --- | --- |
|  |  |  | Univariable analysis | | |  | Multivariable analysis | | |  | Univariable analysis | | |  | Multivariable analysis | | |
| Characteristics |  |  | HR | 95% CI | p value |  | HR | 95% CI | p value |  | HR | 95% CI | p value |  | HR | 95% CI | p value |
| Age | ≥70 |  | 1.2 | 0.70-2.18 | 0.4620 |  |  |  |  |  | 1.5 | 0.75-3.03 | 0.2479 |  |  |  |  |
| Sex | Male |  | 3.3 | 1.70-6.29 | 0.0004 |  | 2.2 | 1.10-4.41 | 0.0260 |  | 3.2 | 1.45-7.19 | 0.0041 |  |  |  |  |
| Smoking | Smoker |  | 3.5 | 1.78-6.84 | 0.0003 |  |  |  |  |  | 4.4 | 1.83-10.8 | 0.0010 |  |  |  |  |
| Pulmonary comorbidity | Present |  | 5.3 | 1.84-15.16 | 0.0020 |  |  |  |  |  | 11.8 | 3.83-36.58 | <0.0001 |  |  |  |  |
| Surgical procedure | ≥Lobectomy |  | 0.6 | 0.33-1.01 | 0.0551 |  | 0.5 | 0.29-0.91 | 0.0225 |  | 0.4 | 0.18-0.74 | 0.0051 |  | 0.3 | 0.15-0.64 | 0.0015 |
| pT | T1b |  | 0.8 | 0.41-1.60 | 0.5481 |  |  |  |  |  | 1.1 | 0.50-2.33 | 0.8515 |  |  |  |  |
| Histological type | Adenocarcinoma |  | 0.2 | 0.12-0.38 | <0.0001 |  | 0.4 | 0.21-0.83 | 0.0117 |  | 0.2 | 0.09-0.37 | <0.0001 |  | 0.3 | 0.12-0.55 | 0.0004 |
| Vascular invasion | Positive |  | 3.8 | 2.00-7.19 | <0.0001 |  | 2.3 | 1.12-4.58 | 0.0236 |  | 2.5 | 1.06-5.68 | 0.0366 |  |  |  |  |
| Lymphatic invasion | Positive |  | 1.6 | 0.39-6.67 | 0.5100 |  |  |  |  |  | 19e^9^ | NA | 0.9993 |  |  |  |  |
| ΔAlb | Decreased |  | 3.6 | 2.03-6.31 | <0.0001 |  | 2.9 | 1.63-5.22 | 0.0003 |  | 4.3 | 2.13-8.55 | <0.0001 |  | 4.3 | 2.09-9.05 | <0.0001 |
| preAlb | Low |  | 2.0 | 1.08-3.59 | 0.0271 |  |  |  |  |  | 3.2 | 1.59-6.44 | 0.0011 |  | 3.4 | 1.65-7.07 | 0.0010 |

HR, hazard ratio; CI, confidence interval; pT, pathological T status; ΔAlb, postoperative decrease in serum albumin; preAlb, preoperative serum albumin levels; NA, not appropriate
